# The Role of Hypofractionated Radiation Therapy with Photons, Protons, and Heavy Ions for Treating Extracranial Lesions

**DOI:** 10.3389/fonc.2015.00302

**Published:** 2016-01-11

**Authors:** Aaron Michael Laine, Arnold Pompos, Robert Timmerman, Steve Jiang, Michael D. Story, David Pistenmaa, Hak Choy

**Affiliations:** ^1^Department of Radiation Oncology, University of Texas Southwestern Medical Center, Dallas, TX, USA

**Keywords:** hypofractionation, photon therapy, ion beam therapy, SBRT, SABR

## Abstract

Traditionally, the ability to deliver large doses of ionizing radiation to a tumor has been limited by radiation-induced toxicity to normal surrounding tissues. This was the initial impetus for the development of conventionally fractionated radiation therapy, where large volumes of healthy tissue received radiation and were allowed the time to repair the radiation damage. However, advances in radiation delivery techniques and image guidance have allowed for more ablative doses of radiation to be delivered in a very accurate, conformal, and safe manner with shortened fractionation schemes. Hypofractionated regimens with photons have already transformed how certain tumor types are treated with radiation therapy. Additionally, hypofractionation is able to deliver a complete course of ablative radiation therapy over a shorter period of time compared to conventional fractionation regimens making treatment more convenient to the patient and potentially more cost-effective. Recently, there has been an increased interest in proton therapy because of the potential further improvement in dose distributions achievable due to their unique physical characteristics. Furthermore, with heavier ions the dose conformality is increased and, in addition, there is potentially a higher biological effectiveness compared to protons and photons. Due to the properties mentioned above, charged particle therapy has already become an attractive modality to further investigate the role of hypofractionation in the treatment of various tumors. This review will discuss the rationale and evolution of hypofractionated radiation therapy, the reported clinical success with initially photon and then charged particle modalities, and further potential implementation into treatment regimens going forward.

## Introduction

After the discovery of X-rays in 1895 and radioactivity in 1896, initial radiation cancer treatments were mostly hypofractionated. Treatments were limited in giving higher doses to the skin and superficial structures than to a deeper tumor target. Quality assurance measures were lacking to ensure accurate dose deposition. These approaches lead to tumor responses, however, with significant late tissue effects. Despite these shortcomings, hypofractionation remained the primary treatment schedule due to patient convenience and technical considerations with treatment delivery.

Early radiotherapy pioneers, including Friedrich Dessauer, sought to address the limitations with the state of technology for delivering hypofractionated treatments. In 1905, Dessauer proposed that clinical outcomes could be improved with the application of homogeneous dose to the tissue and eventually leading to the formulation of ideas of multibeam or multisource irradiation (1).

At the same time, Claudius Regaud was performing his seminal experiments relating to the irradiation of the testis. He observed that cells undergoing mitosis were more sensitive to radiation, whereas the more differentiated cells were less sensitive (2). This work lead to the “Law of Bergonie and Tribondeau” stating that the effects of irradiation on cells are more intense, the greater their reproductive activity, the longer their mitotic phases, and the less differentiated, forming the biological basis for fractionation (3).

By the 1920s, despite the advocacy of Regaud, Antoine Béclère, and Henri Coutard, multiple fractionated treatments were still less popular than hypofractionated treatments. The approach promoted by Ludwig Seitz and Hermann Wintz, favoring intensive short courses of radiotherapy for treatment of cervical cancer, was widely adopted (4).

In 1932, Henri Coutard presented his landmark findings at the American Congress of Roentgenology, demonstrating that protracted-fractionated external beam therapy had cured deep tumors with significantly less toxicity previously seen (5). From this point forward, radiation oncologists across the world mostly abandoned hypofractionated as a method for curative treatment. Interestingly, Coutard believed in both approaches stating that choice of fractionation should depend on the initial volume of the target (small targets warrant hypofractionation, whereas large should be more protracted) (6).

It took until the 1950s, when Lars Leksell, a neurosurgeon, broke from the perceived wisdom of conventionally fractionated radiotherapy (CFRT) by using large-dose single sessions of radiation delivery in the central nervous system (7). In conjunction with a radiation physicist, Borge Larsson, they created the first Gamma Knife (Elekta AB, Stockholm, Sweden). Although a single large-dose radiation treatment was historically intolerable, Leksell’s approach defied conventional wisdom by its technology and conduct. Unlike CFRT, which often irradiates much larger volumes of normal tissue to the prescription dose than the tumor itself if a limited number of beams are utilized, Leksell’s stereotactic radiosurgery surgery (SRS) went to great lengths to avoid delivering high dose to non-targeted tissues. Whatever normal tissue was included, either by being adjacent to the target or by inferior dosimetry, was likely damaged. However, if this damaged tissue was small in volume or non-eloquent, the patient did not suffer clinically apparent toxicity, even as a late event.

Building upon these results, investigators in Sweden at the Karolinska Institute in Sweden by Lax and Blomgren, departed from the established traditions of CFRT and began to explore the use of alternative hypofractionated radiation treatment regimens for the lung, liver, and selected other malignant extracranial tumors. Furthermore, technical advancements in linear accelerators allowing for the delivery of increased beam energies made deep-seeded tumors more accessible with less toxicity. They constructed a stereotactic body frame that would simultaneously enable comfortable and reliable immobilization and dampening of respiratory motion, treating patients with extracranial, localized tumors with ablative doses of radiation that ranged from 7.7 to 45 Gy in 1–4 fractions (8). At the same time in Japan, Uematsu and colleagues developed technologies to deliver stereotactic radiation to lung tumors (9). Initially, the treatments were called extracranial stereotactic radioablation, and later stereotactic body radiation therapy (SBRT) (10, 11). More recently, the descriptive term stereotactic ablative radiotherapy (SABR) has been used (12).

### Radiobiologic Modeling of High Dose per Fraction with Photons

Classical understanding of the mechanisms of radiation induced tumor cell killing centers on the hypothesis that DNA is the main target of ionizing radiation, leading to single- and double-strand breaks. Different mathematical models have been developed to compare tumor control and normal tissue toxicity profiles for various radiation schedules and fraction sizes. The most commonly used is the linear quadratic (LQ) model, which describes cell killing as a single hit versus double hit hypothesis, where the linear cell kill is expressed by the α component, while the quadratic cell kill is expressed by the β component (13). The α/β ratio is obtained from isoeffect curves using the survival fractions of a cell line at different doses per fraction (14). This ratio is primarily utilized to predict the clinical effects in response to changes in fraction size. With regard to tumors, a high α/β ratio predicts higher sensitivity to CFRT, while a lower α/β ratio predicts lower sensitivity to CFRT. Most tumors typically possess a high α/β ratio (~8–10) relative to most normal tissues, which demonstrate lower α/β ratios (~1–4).

Not all hypofractionated radiotherapy is ablative. In general, ablation occurs at dose levels that correspond to the exponential (linear region on a logarithmic scale) portion of the cell-survival curve, which would generally involve daily dose levels of >8 Gy. Below this dose range, cells have more capacity to repair. The logarithm of cell survival as a function of dose in the lower-dose region exhibits a curviness called the shoulder. More conventional and non-ablative hypofractionated radiotherapy is delivered on the shoulder. The range of 2.25–8 Gy per fraction, still considered hypofractionated, has mostly been used for palliation of metastatic disease. More recently, though, investigators treating common diseases, such as breast and prostate cancer, have used non-ablative hypofractionation in patients with curable tumors. This was partly for the cost savings associated with fewer overall fractions, but in some cases such hypofractionation has a biological rationale for improving the therapeutic ratio. A summary of the degrees of hypofractionated radiotherapy is shown in Table 1.

**Table 1:** **Various daily fractionation options**.

Fractionation regimen	Typical dose per fraction (Gy)
Conventionally fractionated radiotherapy	1.5–2.0
Hypofractionated radiotherapy	>2.0–8.0
Ablative radiotherapy	>8.0

Biological effective dose (BED) quantifies the true biological dose delivered by a particular combination of dose per fraction and total dose to a certain tissue characterized by a specified α/β ratio. Based on experimental and clinical data, the LQ model seems to predict BED accurately for fraction sizes <3.25 Gy (15). Due to the fact that typical doses for SBRT/SABR fall outside of this range, the LQ model breaks down as it does not accurately predict the BED for abbreviated hypofractionated regimens (15–18). Development of more accurate models to predict the responses of tumors to hypofractionated radiotherapy have been attempted. The universal survival curve, modified linear quadratic model (LQL), and the generalized LQ model all have shown better radiobiological modeling of high dose per fraction than the LQ model, with moderate success at maintaining accuracy within the conventionally fractionated range (15, 17, 19). These models primarily predict the tumor control to hypofractionated radiotherapy; however, better estimation of normal tissue toxicity with larger doses per fraction is required.

Limitations to predict clinically relevant endpoints exist in simple radiobiological modeling due to the presence of additional factors, including dose rate, period of time over which treatment is delivered, tissue type irradiated, and competing cell death mechanisms besides DNA damage. These may include immunological activation mediated by the release of antigens, damage to cell membranes and organelles, and additional mechanisms related to ablative therapy (20).

Several groups have described tissues and their radiation response according to the organization of the smallest functional subunit (21, 22). Structurally defined tissues can only repair radiation damage by recruiting their own stem cells and have a lower radiation tolerance per functional subunit. Generally, organs comprised such structurally defined subunits, also called parallel functioning tissues, and are large organs, such as the peripheral lung and liver. Parallel organs display significant redundancy in the number of subunits performing the same function to overcome the poor tolerance to damage. By contrast, tissues made up of predominately of structurally undefined subunits are much more tolerant of radiation damage per subunit because of their ability to recruit clonogenic cells from neighboring tissues for repair. Organs made up of structurally undefined subunits, such as the esophagus, major ducts and airways, and spinal cord, are referred as serially functioning tissues and perform critical functions acting as a conduit. Despite possessing a higher radiation tolerance, if a section of a serially functioning tissue is damaged anywhere along its length, all downstream function may be affected (23). The potential to elicit such tissue injury when utilizing ablative doses is a major consideration needed to be taken into account when developing treatment plans.

### Technical and Safety Considerations of Ablative Therapy with Photons

Abbreviated hypofractionated treatments require highly conformal dose distributions that fall off very rapidly in all directions, which require the use of multiple shaped beams (24, 25). Most modern SBRT/SABR treatments utilize 10–12 highly collimated beams or multiple conformal arcs. Effort should be made to create truly isotropically decreasing dose gradients around targets, within the limitations due to potential collisions between the patient or couch and the accelerator head.

The gross tumor volume should be derived by incorporating advanced imaging techniques to assist in the differentiation between tumor and adjacent normal tissue. The planning treatment volume comprises the final target for high-dose conformal coverage and includes an accounting for organ motion. Limitation of the high- and intermediate-dose spillage should be attempted with careful determination of the volume they incorporate. Such spillage should be prioritized to avoid potential adjacent serially functioning tissues to reduce potential injury and downstream effects.

In summary, the defining characteristics of SBRT/SABR include the following (23): (1) secure immobilization avoiding patient movement for the typical long treatment sessions; (2) accurate repositioning from simulation to treatment; (3) minimization of normal tissue exposure attained by using multiple (e.g., 10 or more) or large-angle arcing small aperture fields; (4) rigorous accounting of organ motion; (5) stereotactic registration (i.e., via fiducial markers or surrogates) of tumor targets and normal tissue avoidance structures to the treatment delivery machine; and (6) ablative dose fractionation delivered to the patient with subcentimeter accuracy.

## Clinical Results of Hypofractionation with Photons

### Non-Small Cell Lung Cancer

For patients with medically inoperable non-small cell lung cancer (NSCLC), dose escalation using conventional fractionation was initially explored to improve the probability of local control. Radiation Therapy Oncology Group (RTOG) Protocol 7301 investigated multiple dosing regimens for patients with T1-3 N0-2 disease, including 40 Gy delivered in a split regimen of two courses of 20 Gy delivered in 5 fractions (40 Gy total in 10 fractions) with a 2-week break between courses, and continuous regiments escalating the dose from 40 to 60 Gy. The failure rate within the irradiated volume was 48% in the 40 Gy continuous regimen, 38% for the 40 Gy split course and 50 Gy regimen, and 27% in the 60 Gy continuous regimen (26). RTOG Protocol 9311 then escalated doses from 65 to 90.3 Gy using 3D conformal radiation therapy in inoperable patients, and found that treatment could safely be delivered in daily fraction sizes of 2.15 Gy to a total dose of 77.4 Gy, or 83.8 Gy provided that the volume of lung receiving 20 Gy could be constrained to less than 25% of the total lung volume. The study attained locoregional control rates at 2 years of 55–78% at the maximum tolerated dose (MTD) (27).

In order to continue to improve LC and OS in this patient population, protocols have sought to improve the therapeutic ratio with the addition of chemotherapy or by changing the dose per fraction. Researchers at Indiana University reported a Phase 1 study in which patients with T1–T2 N0 NSCLC were treated with escalating doses of SBRT/SABR, starting at 24 Gy in three fractions and increasing to 60 Gy (for T1 lesions) or 72 Gy (for T2 lesions) in three fractions to determine the MTD. The MTD was not reached for T1 lesions at 60 Gy, and for T2 lesions an MTD of 66 Gy was established based on bronchitis, pericardial effusion, hypoxia, and pneumonitis. Crude rates of local failure were 21% in both the T1 and T2 cohorts, and a dose–response was noted with only one local failure observed with fraction sizes of >16 Gy per fraction (10, 28). These doses were calculated without correction for tissue inhomogeneity; subsequent doses used inhomogeneity correction and, as a result, appear slightly lower.

A subsequent Phase 2 multicenter trial (RTOG 0236) further evaluated the toxicity and efficacy of SBRT in a high-risk population of patients with T1-2aN0 (lesions <5 cm in size) early stage, medically inoperable NSCLC. Doses of 54 Gy in three fractions were delivered, and an estimated 3-year local control rate of 97.6% was observed, with an overall survival rate of 55.8% at 3 years (29). Based on this study, SBRT/SABR is now the standard of care for medically inoperable early-stage NSCLC or those patients who refuse surgery. Further work is being done to optimize dose delivery for early stage NSCLC; the RTOG conducted RTOG Protocol 0915, a randomized Phase II study that compared two different SBRT/SABR treatment schedules for medically inoperable patients with Stage I peripheral NSCLC, in which patients were randomized to receive 34 Gy in a single fraction or 48 Gy in four daily consecutive fractions of 12 Gy per fraction. This protocol is now closed to accrual, and final results are pending; preliminary data suggest that 34 Gy may be more efficacious with respect to local control and equivalent in toxicity profile, and a comparison of 34 Gy in one fraction to 54 Gy in three fractions is planned.

### Primary Liver Cancer

In a phase I feasibility trial at Indiana University, patients with hepatocellular carcinoma (HCC) were treated with dose escalation from 36 Gy in three fractions to a total dose of 48 Gy in three fractions if dose-limiting toxicities were not suffered (30). Patients were eligible for this trial if they had Child–Pugh score A or B, a solitary tumor <6 cm in size or three lesions with total diameter <6 cm and adequate liver function. Key normal tissue constraints were that one-third of the uninvolved liver received ≤10 Gy for Child–Pugh class A patients and that one-third of the uninvolved liver received ≤15 Gy for Child–Pugh class B patients. In this study, the dose was successfully escalated to patients with Child–Pugh class A to 48 Gy in three fractions without reaching dose-limiting toxicity. However, in patients with Child–Pugh class B cirrhosis, the maximum tolerated dose was 40 Gy in five fractions due to two patients suffering Grade 3 liver toxicity. With long-term follow-up, the Indiana experience found positive rates of 2-year local control of 90% among the treated population. There were no long-term grade 3 or higher non-hematologic toxicities and 20% of patients were found to experience progression in the Child–Pugh score at 3 months (31).

A second key phase I/II trial was performed by Princess Margaret University and the University of Toronto (32). In this trial, patients with Child–Pugh score A with no more than five liver tumors with a maximal dimension of 15 cm were enrolled. Patients in this trial were treated to a dose of 30 to 54 Gy in six fractions, with the maximum effective irradiated liver volume of 60%. No patients in this trial suffered classic radiation-induced liver disease (RILD) or dose-limiting toxicity, with a decline in Child–Pugh score at 3 months occurring in 29% of the cohort. Like the Indiana experience, the local tumor control was excellent at 87% at 1 year. These two trials provide data for the efficacy for SBRT/SABR in the setting of well-controlled and designed clinical trials.

While these studies were limited to patients with preserved to mildly elevated liver function, there is evidence for the treatment of patients with Child–Pugh B7 or B8 with SBRT/SABR as well. The Princess Margaret group performed a prospective study with patients having Child–Pugh B7 or 8 with less than 10 cm of HCC tumor (33). Patients received a median dose of 30 Gy in five fractions; however, as expected with their more fragile liver function, 63% of the cohort had a decline in their Child–Pugh score at 3 months. Sorafenib is a tyrosine kinase inhibitor that was used in patients with advanced HCC, showing an improvement in overall survival compared to placebo. Currently, an RTOG trial 1112 is enrolling patients with advanced stage HCC to daily sorafenib versus SBRT/SABR alone followed by daily sorafenib. The primary endpoint of the trial is overall survival with secondary endpoints, evaluating the safety profile of SBRT/SABR plus sorafenib. This trial will potentially further expand the utilization of SBRT/SABR patients with advanced HCC.

### Prostate Cancer

Recent analysis and review of clinical outcomes, primarily after treatment with brachytherapy, argue for a low α/β for prostate cancer of ~1.5 (34–38). Several recent clinical trials were designed with the explicit assumption of this low α/β ratio by utilizing more hypofractionated regimens in comparison with conventional schedules (39–44). Altogether, these trials show that the treatment can be delivered much more quickly and conveniently using equivalent effective doses with hypofractionation without compromising PSA control or significant toxicity so long as careful technique and normal tissue dose tolerance is respected. Building upon this premise, even more abbreviated hypofractionated approaches (6.5–10 Gy per fraction) have been investigated.

The Virginia Mason Medical Center published one of the first experiences with prostate SBRT/SABR, describing their results from a phase I/II trial delivering 33.5 Gy in five fractions (45). Median follow-up was 41 months. There was one acute grade 3 urinary toxicity (urinary retention requiring catheterization) and no acute grades 4–5 toxicities. Late grade 2 GU and GI toxicity rates were 20 and 7.5%, respectively, with no grade 3 or higher toxicities. Four-year actuarial freedom from biochemical recurrence (FFBR) was 90%.

The feasibility of increasing SBRT/SABR dose was investigated at Stanford University in a phase II trial (46). 36.25 Gy in five fractions of 7.25 Gy was delivered to the prostate plus a 3–5 mm margin. In 67 patients with low to intermediate-risk features (Gleason score 3 + 3 or 3 + 4, PSA ≤10 ng/mL, and clinical stage ≤T2b), there were no grade 4 or higher toxicities. Late grades 2 and 3 GU toxicity rates were 5 and 3.5%, respectively. Late grade 2 GI toxicity was 2% with no grade 3 or higher toxicities seen. Patients who received QOD treatments were less likely to experience grades 1–2 GI and GU toxicities than those who received QD treatments. Four-year PSA relapse-free survival was 94%.

The largest prospective study of prostate SBRT/SABR is from the Winthrop University Hospital (47). A total of 304 patients (69% low-risk, 27% intermediate-risk, 4% high-risk) were treated. The first 50 patients received 35 Gy in five fractions of 7 Gy with the subsequent 254 patients receiving 36.25 Gy in five fractions of 7.25 Gy. Lower-dose patients had a median follow-up of 30 months and the higher-dose patients had a median follow-up of 17 months. There were no grades 3–4 acute complications. Late grade 2 GU and GI toxicity was 14 and 7%, respectively. Five patients had late grade 3 GU toxicity with no late grades 4–5 toxicities. For patients who were potent prior to treatment, 75% stated that they remained sexually potent. Actuarial 5-year biochemical recurrence-free survival was 97% for low-risk, 90.7% for intermediate-risk, and 74.1% for high-risk patients.

A recent pooled analysis of 1100 patients from prospective phase II trials using SBRT/SABR for the treatment of prostate cancer in which a median dose of 36.25 Gy was delivered in four to five fractions demonstrated a 93% 5-year biochemical relapse-free survival rate for all patients (95% for low-risk, 84% for intermediate-risk, and 81% for high-risk) with favorable long-term patient reported outcomes with respect to urinary and bowel functions (48, 49).

Compared to the prior studies using similar dose fractionation regimens, a multicenter phase I/II trial investigation using significantly higher doses was performed (50). In the phase I portion, 45 patients, in 3 cohorts of 15, were treated with 45, 47.5, and 50 Gy in five equal fractions, respectively. No dose-limiting toxicities (grades 3–5) occurred within the first 90 days post-treatment. GI grade ≥2 and grade ≥3 toxicity occurred in 18 and 2%, respectively, and GU grade ≥2 and grade ≥3 toxicity occurred in 31 and 4%, respectively. Initial PSA control was 100%. These encouraging results led to the further enrollment on the phase II trial at the 50 Gy dose level studying late toxicity. An additional 46 patients were enrolled for a total of 91 (64% intermediate-risk and 36% low-risk). With a median follow-up of 42 months, PSA control remained at 99% (51). Ultimately, dose escalation to treat prostate cancer is limited by toxicity to the bladder or rectum. As reported in an update by Kim et al., the toxicity profile was favorable in the initial phase I results; however, in the phase II portion, the profile changed and five patients (10.6%) developed high-grade rectal toxicity (52).

## Hypofractionation with Proton and Heavier Ion Therapy

### Background

Protons and other heavier charged particles offer some theoretical advantages over photons that can be utilized in hypofractionated dose delivery regimes.

As charged particles move in tissue, they deposit energy (dose) and cause ionization of tissue and create highly reactive free radicals. This has two important consequences. One is that after traversing tissue for a certain depth, they impart all of their initial kinetic energy and they stop moving. In other words, charged particles have a finite range in tissue, unlike a photon beam, that can only be exponentially attenuated but not stopped. The second consequence is that these free radicals cause biological damage. As more dose is deposited, more ionization occurs generating more free radicals, leading to a higher biological damage. The amount of absorbed dose per unit track length [called the linear energy transfer (LET) to tissue] increases as they lose speed along their paths. At first, very gradually in the tissue entrance region where particles are moving with speed close to the speed of light, but then very rapidly toward the end of their range where they substantially slow down so that a peak of deposited dose occurs at a depth proportional to the initial kinetic energy of the charged particle. Beyond this peak, no further significant dose is deposited. This scientific phenomenon was described and experimentally discovered by William Bragg at that time (53). As mentioned above, the range in tissue is proportional to initial kinetic energy of the particles. Hence, particle accelerators are needed to get the initial speed high enough so they reach even deep seeded tumors. In 1930, the American physicist Ernest O. Lawrence and his associates were the first to invent a cyclotron to accelerate protons. Since then, the technology has substantially improved and many proton cyclotrons and particle synchrotrons have been built to reach energies high enough for cancer treatment applications.

The idea to use proton and charged particle beams for cancer treatment came in 1946, when Wilson wrote his seminal paper (54). He realized that the fundamental difference in dose as a function of depth (depth–dose curve) of proton and heavy-charged particles, in comparison with photons, can be used to spare healthy tissue at the tissue entrance region where smaller amount of energy is released, but to achieve high tumor control at the peak, where much larger amount of the beam energy is being released. Also healthy tissue can be spared beyond where no protons are present since they already stopped at the peak region. The first proton patient was treated in 1954 at Lawrence Berkeley National Laboratory (LBNL) nuclear research facility and the first clinical center to use proton-based therapy was based at Loma-Linda and initially used to treat pituitary hormone suppression in metastatic breast carcinoma.

Ions heavier than a proton were first used for cancer therapy in the 1970s after Cornelius Tobias hypothesized that they could provide additional clinical advantages (55). Heavier ions have reduced lateral scattering compared with protons. This translates into faster lateral dose fall off (called sharper lateral penumbra) leading to a better ability to conform dose to the target region; hence, higher therapeutic doses can be prescribed to tumors located in near proximity to radiosensitive organs at risk. Another huge advantage of heavier ions is that they interact with tissue they create a small amount of radioactive positron emitting isotopes that can be imaged in PET/CT scanners providing direct *in vivo* information about the spatial distribution of delivered dose. The higher ionization density created by heavier ions leads to their increased radiobiological effects on tissues. Initial radiobiology research and clinical treatment used beams of nuclei of helium, carbon, neon, silicon, and argon atoms; however, most of the clinical experiences with ions heavier than protons involves carbon, because this particle has approximately the same biological potency as photons or protons in the tissue entrance region and three to four times larger potency in the Bragg peak region even if the same dose was absorbed (56).

### Radiobiological Modeling of Hypofractionation with Protons and Heavier Ions

A generalized statement is that the higher the electric charge of the charged particle, the higher the energy loss per unit track length (quadratically higher) while penetrating tissue. Therefore, the LET is 36 times higher for carbon ions compared with protons when they move with the same speed (57). The increased LET has a consequence of increased biological potency expressed by relative biological effectiveness (RBE) that is defined as a quotient describing how many times more dose is needed to be delivered by photons than by charged particles to achieve the same biological endpoint. Clinical proton beams are of low LET with a RBE very close to that of high-energy photons. Recently, the International Commission on Radiation Units and Measurement (ICRU) proposed a RBE of 1.10 regardless of depth in tissue for proton therapy (58). The RBE of heavier ions to be clinically used is still under investigation (59–61).

As mentioned above, another difference between protons and carbon is that the RBE for carbon ions varies, with an increase in the Bragg peak region. This increase in RBE needs to be accounted for in treatment planning in order to have an appropriate prescribed dose (59, 62, 63). Calculation of the RBE is complex in that it depends on multiple factors, including the particle species, ion beam energy, dose, tissue type being irradiated, and biological endpoint. An accurate description of RBE dependence on dose is even more critical in the setting of hypofractionation (64–66). Recently, an excellent review of how variation of the RBE of ion beams in the setting of hypofractionated radiotherapy was presented by Friedrich et al. (60). In general, increasing the dose per fraction leads to lower RBE of the tumor and normal tissue (67). Data suggest that the RBE of the tumor decreases more slowly than the RBE of the normal tissue (68, 69). Therefore, hypofractionated heavy-ion treatment can be used to spare the organs at risk while escalating dose to the tumor.

### Technical Considerations of Hypofractionation with Protons and Heavier Ions

Very tight prescription dose conformity to the target and very sharp dose fall off between tumor tissue and healthy tissue are essential for hypofractionation approach to succeed. As described in Section “Background,” the physics of heavy-charged particle interactions with tissue theoretically offers superior solutions to achieve both of these goals with respect to photons. It is the beam delivery techniques that differ between photons and charged particles. Heavy-charged particle therapy physics and engineering has yet to develop the most advanced therapy, namely the equivalent of the volumetric photon arc therapy that would fully utilize the physics advantages of heavier charged particles.

Most particles centers are currently using scattering techniques with energy modulators, patient-specific collimators, and compensators to spread out protons and carbon ions both in longitudinal and lateral directions for treatment. Scattering beam delivery is easier for planning and quite robust in the treatment of a moving target. There are two major downsides of this technique. It requires the usage of patient and beam direction-specific collimators and compensators, which limits the number of irradiation directions that could practically be feasible. Another downside of this approach is that the dose to the preceding normal tissue in the entrance path is very difficult to modulate and conform to the target. It is, therefore, higher compared with modern beam scanning techniques, leading potentially to an increased risk in the development of secondary malignancies and other healthy tissue toxicities. Recently, more active beam delivery techniques, spot scanning or raster scanning, have been developed. Spot scanning is superior to passive beam delivery in terms of the improved dose profile and the reduced amount of material in the beam line, which decreases the leakage or radiations and neutrons (56). However, the capability to treat moving targets with scanning beams remains a challenge since longitudinal (in the direction of energy) beam modulation is still relatively slow. Approaches, such as target motion tracking, have been proposed (70) with systems capable of fast energy change of individual spots. Real-time detection of tumor and surrounding organ motion is vital for such a technique to succeed and remains a problem.

Another important consideration is how to deliver multidirectional particle beams. As mentioned above, highly conformal rapid dose fall off that is achieved with SABR photon therapy relies on the utilization of multiple beam angles. If the same is used with heavy-charged particles, both high-dose and intermediate-dose volume regions outside of the targeted area can substantially be reduced. Furthermore, with photons, the size of tumor targets (radius, *r*) to which high dose hypofractionation can be applied is limited to 2–3 centimeters in diameter. This is due to the fact that even for relatively small (Δr) region of high dose over spillage, the volume receiving the high dose grows quadratically with *r* and linearly with Δr. Doubling the target size “*r*” would require to reduce the high dose over spillage Δr by factor of 4 to keep the same volume of high dose over spillage. This is impossible to achieve with photons, but heavier charged particles have the Δr intrinsically much lower than photons both in front, beyond, and lateral to tumors.

Currently, many heavy-ion centers used fixed angle beams from different directions and tilt the patient’s couch to provide different entrance angles, but multiple CT scans and treatment plans are necessary and the magnitude of patient tilt is limited. The use of a rotating gantry would allow for a beam delivery from any angle. Currently, the first heavy-ion rotating gantry is in use at the Heidelberg Ion Therapy Center (HIT) in Germany and another is currently under construction at the National Institute of Radiological Sciences (NIRS) in Japan. Further results from these centers should help to shed light on if the clinical advantages live up to the theoretical dosimetric advantages listed above.

### Clinical Results of Hypofractionation with Protons and Heavier Ions

The higher conformal beam delivery with particles, compared to photons, allows for dose escalation to the tumor without exposing the adjacent organs at risk to higher doses (56, 71). Compared with photon therapy, it is assumed that using particles results in a lower integral dose to normal tissues and a lower whole-body neutron exposure (72). To date, more hypofractionated approaches have been utilized in carbon-ion therapy compared to proton therapy, where more conventional fractionation regimens are employed (57). Additionally, most of the clinical data on proton and carbon therapy were collected from patients treated with passively scattered beams. More recently, active scanned proton and carbon-ion beams have been developed and used for clinical treatment.

#### Non-Small Cell Lung Cancer

For early stage I peripheral NSCLC tumors, local control rates are high for hypofractionated photons. The use of protons and carbon ions for the treatment of early stage tumors have been studied to avoid lung toxicity by sparing normal lung tissue while facilitating escalated dose to the tumor.

At Loma Linda University, medically inoperable patients with clinical T1–T2, N0, M0 NSCLC were treated with hypofractionated proton therapy (73). The dose delivered was escalated from 51 to 60 GyE, then to 70 GyE in 10 fractions over 2 weeks. Four-year local control for T1 tumors treated with either 60 or 70 GyE were 86 and 91%, respectively. Decreased control rates were seen for T2 tumors, 45 and 74%, respectively. Good outcomes were seen for patients with peripheral T1 tumors, with 4-year local control of 96%, disease-specific survival of 88%, and overall survival of 60%. No treatment-related adverse events of grade 2 or higher were observed.

Nihei et al. treated 37 inoperable patients with Stage I NSCLC (74). A total dose of 70 to 94 GyE was delivered in 20 fractions in 4–5 weeks. Two-year local control rates for Stages 1A and 1B tumors were 100 and 90%, respectively. Three patients (8%) experienced grade 3 pulmonary toxicity.

Hata et al. treated 21 patients (11 with Stage 1A and 10 with Stage 1B) NSCLC were treated with 50 GyE (three patients) or 60 GyE (18 patients) in 10 fractions over 15 days (75). Two-year local control rates were 100% for Stage 1A and 90% for Stage 1B, respectively. The overall and cause-specific survival rates in all patients were 74 and 86% at 2 years, respectively. No grade 3 or higher toxicities were observed.

The initial dose-escalation experience with treating Stage I NSCLC tumors with carbon ions was reported by Miyamoto et al. (76). The first stage phase I/II trial using 18 fractions over 6 weeks for 47 patients and the second one using nine fractions over 3 weeks for 34 patients were conducted by the dose-escalation method from 59.4 to 95.4 GyE in incremental steps of 10% and from 68.4 to 79.2 GyE, respectively. The local control rates in the first and second trials were 64 and 84%, respectively. The doses greater than 86.4 GyE at 18 fractions and 72 GyE at nine fractions achieved a local control of 90 and 95%, respectively. Grade 3 radiation pneumonitis occurred in three of 81 patients, but they fully recovered.

Building upon this experience, Miyamoto et al. further treated 29 Stage 1A and 21 Stage 1B patients who were treated with 72 GyE in nine fractions over 3 weeks (77). There was 1 in-field (Stage 1A) and 1 margin (Stage 1B) failure. Two- and 5-year actuarial local control rates were 98 and 95%, respectively. There was one grade 3 late skin reaction. A further phase II study using a regimen of a fixed dose of 52.8 GyE for T1 tumors and 60 GyE for T2 tumors in four fractions over 1 week was performed (78). The local control rate at 5 years for all patients was 90% (T1: 98%, T2: 80%). No grade 3 or higher toxicities were seen. Currently, a dose-escalation study for single-fraction treatment is underway at the NIRS, where initial results have shown higher local control and survival rates with minimal toxicity (79). A comparison of the outcomes of treating NSCLC with different modalities is shown in Table 2.

**Table 2:** **Non-small cell lung cancer**.

Reference	Radiation	Dose GyE	Dose/Fx GyE	Stage	*n*	Local control (%)	Late toxicity ≥grade 3
Timmerman et al. (10)	X	60	20	T1	70	89 at 3 years	10% peripheral
		66	23	T2			27% central
Timmerman et al. (29)	X	54	18	T1–2a	59	98 at 3 years	15%
Baba et al. (80)	X	44–52	11–13	T1–2	124	80 at 3 years	3%
Bush et al. (73)	P	51–60	5.1–60	T1	68	86 at 4 years	None
				T2		45 at 4 years	
Nihei et al. (74)	P	70–94	3.5–4.9	T1a	37	100 at 2 years	None 15%
				T1b		90 at 2 years	
Hata et al. (75)	P	50–60	5–6	T1a	21	100 at 2 years	None
				T1b		90 at 2 years	
Miyamoto et al. (76)	C	59.4–95.4	3.3–5.3	T1–2	81	76 at 5 years	None
		68.4–79.2	7.6–8.8				
Miyamoto et al. (77)	C	72	8	T1a–b	50	95 at 5 years	2%
Miyamoto et al. (78)	C	52.8	13.2	T1	79	98 at 5 years	None
		60	15	T2		80 at 5 years	

*X, photon therapy; P, proton therapy; C, carbon-ion therapy; Fx, fraction; GyE, gray or gray equivalent; *n*, patient number*.

A recent meta-analysis compared particle beam therapies and SBRT/SABR versus CFRT (81). Five studies with proton therapy and three studies with carbon-ion therapy were included. Due to the limited number of patients available for analysis, no significant results were able to be obtained for locally advanced NSCLC; however, statistical comparisons were able to be made for stage I tumors. They summarized that CFRT had worse overall survival and disease-free survival compared to SBRT/SABR and particle beam therapies. The corrected pooled estimates for 5-year overall survival and DFS rates were 19 and 43% for CFRT, 42 and 63% for SBRT/SABR, 40 and 52% for protons, and 42 and 64%for carbon ions, respectively. Lastly, adverse events appeared to be reduced by using particle therapies compared to photon therapies.

These results document that high 2- to 5-year local control rates of Stage I NSCLC are being achieved by several methods. The carbon-ion therapy 5-year local control results are >95% with minimal toxicity. The 2-year proton local control rates are similar to those by SBRT/SABR but evidently with lesser risk of complications. Longer follow-up is required to assess the clinical efficacy of these three modalities.

#### Liver Cancer

Fukumitsu et al. treated 51 HCC patients with protons to a total dose of 66 GyE in 10 fractions (82). Patients were Child–Pugh class A or B and whose tumors were ≤10 cm (88% ≤5 cm), 39% of patients had multiple tumors and were ≥2 cm from porta hepatitis and GI tract. The 3- and 5-year local control rates were 95 and 88%, respectively. Alpha fetal protein levels dropped from 97 ng/mL before treatment to 16 ng/mL afterwards (*p* < 0.0001). Patients experienced only minor acute reactions of Grade 1 or less, and three patients experienced late sequelae of Grade 2 or higher.

Chiba et al. reported on their results using proton beam therapy to treat 162 patients with HCC with a total of 192 lesions (83). The median total dose delivered was 72 GyE in 16 fractions over 29 days. Eighty-three percentage of the lesions were <5 cm in size. The 5-year local control rate was 87%. Thirteen tumors locally recurred between 7 and 43 months (median, 21 months) after the completion of the irradiation. Maximal diameter of tumors that had recurred was median 4.7 cm ranging from 2.0 to 7.0 cm before irradiation. Five patients had late sequelae of grade 2 or higher.

Bush et al. performed a phase II trial in which 76 patients received 63 GyE in 15 fractions over 3 weeks (84). The mean tumor size was 5.5 cm. Fifteen patients (20%) experienced local treatment failure. Only three patients developed solitary local failure, the majority (36%) developed new lesions within the liver. No patients developed RILD.

Komatsu et al. published a retrospective analysis of 343 patients treated with either protons (242 patients) or carbon ions (101 patients) (85). Eight protocols for proton therapy (52.8–84 GyE in 4–38 fractions) and four protocols for carbon-ion therapy (52.8–76 GyE in 4–20 fractions) were used during the study period. The 5-year local control rate was 90% for proton and 93% for carbon ions. Univariate analysis identified tumor size as an independent risk factor for local recurrence. Grade ≥3 late toxicities were observed in eight patients on proton therapy and in four patients on carbon-ion therapy, and 4 of 12 patients were diagnosed with RILD. No patients died of treatment-related toxicity.

Kato et al. presented results of 24 patients treated on a dose-escalation trial (49.5–79.5 GyE in dose increments of 10% in a fixed 15 fraction setting within 5 weeks) (86). The overall local control rate was 92, 81, and 81% at 1, 3, and 5 years, respectively. No local failures were seen at a dose level of 72 GyE or higher. Except for one early skin reaction, no Grade 3 or worse adverse effects occurred at any dose level from 49.5 to 79.5 GyE. More recently, a four-fraction regimen has been investigated at NIRS. Sixty-nine patients have been treated using this regimen with a reported 5-year local control rate of 81% (79). An accelerated schedule of two fractions in 2 days is being studied further. Another dose-escalation trial is currently underway by Combs et al. at the HIT (87). The Prometheus trial escalates the dose from 40 to 56 GyE in four fractions (87).

A comparison of the outcomes of treating HCC with different modalities is shown in Table 3. At present, the clinical results of protons appear to be equivalent to those of carbon therapy. It will be important to determine the long-term functional status of the liver following dose escalation.

**Table 3:** **Hepatocellular carcinoma**.

Reference	Radiation	Dose GyE	Dose/Fx GyE	Stage	Size (Ave)	*n*	Local control (%)	Late toxicity ≥grade 3
Andolino et al. (31)	X	36–48	13–16	CPC-A	3.2 cm	36	90 at 2 years	35% heme/liver
		40	8	CPC-B		24		
Bujold et al. (32)	X	24–54	4–9	CPC-A	7.2 cm	102	87 at 1 year	30% (7 G5)
Culleton et al. (33)	X	30	5	CPC-B	5.1 cm	28	55 at 1 year	63% ↑CP ≥2
				CPC-C		1		
Fukumitsu et al. (82)	P	66	6.6	CPC-A	2.8 cm	51	88 at 5 years	1 Lung
				CPC-B				
Chiba et al. (83)	P	72	4.5	CPC-A–C	3.8 cm	162	87 at 5 years	3% ≥G2
Bush et al. (84)	P	63	4.2	CPC-A–C	5.5 cm	76	80 (2–60 m)	None
Komatsu et al. (85)	P	52.8–84	2–13.2	CPC-A–C	<5 cm 74%	242	90 at 5 years	3%
	C	52.8–76	3.8–13.2			101	93 at 5 years	4%
Kato et al. (86)	C	49.5–79.5	3.3–5.3	CPC-A–B	5 cm	24	81 at 5 years	25% ↑CP ≥2

*X, photon therapy; P, proton therapy; C, carbon-ion therapy; Fx, fraction; GyE, gray or gray equivalent; *n*, patient number; CPC, Child–Pugh Class*.

#### Prostate Cancer

Proton ± photon therapy was used to treat 1255 patients at Loma Linda University (88). The patients were stage I–IIIA who had no prior surgery or hormone therapy. Radiation dose was 74 GyE in 37 fractions for patients receiving protons only. The 5- and 10-year biochemical no evidence of disease (bNED) was 75 and 73%, respectively. The rate of grades 3–4 rectal and bladder complications was 1%.

The first phase III clinical trial of photons versus protons was conducted by Shipley et al. (89). A total of 189 patients with stages T3–T4 were initially treated with 50.4 Gy by photons to the prostate and pelvic nodes followed by either a photon boost to 67.2 Gy or a proton boost to 75.6 GyE. Patients did not receive any concomitant or adjuvant hormone therapy. The local control at 5 and 8 years for the photon arm were 80 and 60%, respectively, and for the proton arm were 92 and 77%, respectively. Complications in the photon and proton arms at 8 years were as follows: persistent rectal bleeding 2 versus 9%; urethral stricture 2 versus 4%, and hematuria 2 versus 2%.

A phase III trial was performed at MGH and Loma Linda University, which randomly assigned patients with T1b–T2b and PSA≤15 ng/mL to treatment by proton beams to the prostate to 19.8 or 28.8 GyE followed by 50.4 Gy with photons to the pelvis (90). Dose fractionation was 1.8 Gy/fraction for the entire regimen. A total of 393 men were randomized. The 10-year ASTRO biochemical failure rates were 32% for conventional dose and 17% for high-dose radiation therapy. Dose escalation also was shown to benefit patients with low-risk disease. Two percentage of patients in both arms experienced late grade ≥3 genitourinary toxicity, and 1% of patients in the high-dose arm experienced late grade ≥3 gastrointestinal toxicity.

Recently, prospective proton-only trials have been performed at the University of Florida (91). A total of 211 patients were enrolled and received 78 GyE in 39 fractions for low-risk disease (*N* = 89), dose escalation from 78 to 82 GyE for intermediate-risk disease (*N* = 82), and 78 GyE with concomitant docetaxel followed by androgen deprivation for high-risk disease (*N* = 40). With early follow-up of 2 years, progression-free survival was 99% for the entire population (100% for low-risk, 99% for intermediate-risk and 94% for high-risk). Only 1.9% Grade 3 GU symptoms and <0.5% Grade 3 GI toxicities were observed.

Several carbon-ion dose-escalation studies have been performed at the NIRS since 1995. These early trials were summarized by Tsuji et al. (92). The two previous Phase I/II studies performed dose escalation from the initial dose of 54.0 GyE in 20 fractions to 72.0 GyE in 20 fractions in 10% increments followed by fixed radiation dose of 66.0 GyE in 20 fractions. A total of 201 patients were analyzed at the 66.0 GyE dose level with a median follow-up of 30 months. The bNED for the low-risk patients was 100% and for the high-risk patients was 81% at 5 years. In the first Phase I/II study, 6 out of 14 patients treated with a high target dose (72.0 GyE) developed Grade 3 morbidities of the rectum or genitourinary system. At the 66.0-GyE dose level, no Grade 3 or higher toxicities were observed in either the rectum or genitourinary system, and the incidences of Grade 2 rectum or genitourinary morbidity were only 1.0 and 6.0%, respectively.

More recently, a total dose of 57.6 GyE in 16 fractions has been explored (93). A total of 664 patients (250 patients at 66.0 GyE in 20 fractions; 216 63.0 GyE in 20 fractions; 198 in 57.6 GyE in 16 fractions) with at least 1-year follow-up were analyzed in regard to late radiation toxicity. The 5-year biochemical relapse-free survival was 90% for the entire group. The 5-year BRF of patients treated with 16 fractions was 89% compared with 90% for patients treated with 20 fractions. Per risk group, the 5-year BRF was 90, 97, and 88% for low-, intermediate, and high-risk patients (94). No grade 3 or higher GI toxicity was seen in any of the groups and only one grade 3 GU toxicity was seen in the 20 fraction regimen and no grade 3 or higher GU adverse effects were seen in the 16 fraction regimen. Lately, patients have been treated with the scanning beam in the new NIRS facility with 12 fractions in 3 weeks (79). Also the HIT has conducted a randomized phase II trial using protons or carbon ions treating with a 66 GyE in 20 fractions and the results are still pending (95).

A comparison of the outcomes of treating prostate cancer with different modalities is shown in Table 4.

**Table 4:** **Prostate cancer**.

Reference	Radiation	Dose GyE	Dose/Fx GyE	Stage	*n*	Local control or bNED%	Late toxicity ≥grade 3
Madsen et al. (45)	X	33.5	6.7	≤T2a PSA≤10 GS≤6	40	bNED 90% at 4 years	G2 GU 20% G2 GI 7.5%
King et al. (46)	X	36.25	7.25	≤T2b PSA≤10 GS≤7 (3 + 4)	67	bNED 94% at 4 years	GU 3.5% GI 0%
Katz et al. (47)	X	35–36.25	7–7.25	69% low 27% intermediate 4% high	304	bNED 97% at 5 years bNED 91% at 5 years bNED 74% at 5 years	GU 2.5% G2 GI 7%
Boike et al. (50) and Kim et al. (52)	X	45–50 50	9–10 10	≤T2b PSA ≤20 GS ≤7	45 46	bNED 99% at 3.5 years	GU 5.5% GI 6.6%
Slater et al. (88)	P ± X	74	2	Low, intermediate, high	1225	bNED 75% at 5 years	GU, GI 1%
Shipley et al. (89)	X + P X	67.2 75.6	1.9	T3-4, N0-N2, M0	96 93	LC 81% at 5 years LC 92% at 5 years	GU, GI 2% GU 4%; GI 9%
Zietman et al. (90)	X + P	70.2 79.2	1.8 1.8	≤T2b PSA ≤15 GS any	197 196	bNED 61% at 5 years bNED 80% at 5 years	GU 2%; GI 1% GU 1%; GI 1%
Mendenhall et al. (91)	P	78 78–82 78	2 2 2	Low Intermediate High	89 82 40	bNED 100% at 2 years bNED 99% at 2 years bNED 94% at 2 years	GU 1.9% GI < 0.5%
Tsuji et al. (92)	C	54–72 66	2.7–3.6 3.3	≤T3 PSA any GS any	94 201	bNED 92% at 5 years	Six points at 72 GyE None at 66 GyE
Okata et al. (93)	C	63 57.6	3.15 3.6	≤T3 PSA any GS any	216 198	bNED 90% at 5 years bNED 89% at 5 years	1 pt GU at 63 GyE

*X, photon therapy; P, proton therapy; C, carbon-ion therapy; Fx, fraction; GyE, gray or gray equivalent; *n*, patient number; PSA, prostate-specific antigen; bNED, biochemical no evidence of disease; LC, local control; GU, genitourinary; GI, gastrointestinal*.

### Cost-Effectiveness of Hypofractionated Therapy

Due to the aging of the population in industrialized countries, the number of patients who develop malignancies is expected to increase. With more patients receiving cancer-directed therapies, this is predicted to become a major burden for health care systems (96). There has been increasing pressure within the medical community to identify and promote more cost-effective treatment modalities. A significant percentage of the cost of cancer therapy is due to drug therapies (97). This highlights the growing need for a value-based system that considers cost-effectiveness in determining cancer drug prices. There is a lack of correlation between drug efficacy and cost, with prices remaining high despite the entrance of competitive agents to the market (98).

The incremental cost-effectiveness ratio (ICER) is a statistic used in cost-effectiveness analysis to summarize the cost-effectiveness of a health care intervention. It is defined by the difference in cost between two possible interventions, divided by the difference in their effect. It represents the average incremental cost associated with one additional unit of the measure of effect.

Using an ICER approach, actually, radiation therapy is an extremely cost-effective cancer therapeutic option in comparison to systemic modalities. For example, in using improvement in 2-year overall survival as the heath outcome effect, the ICER is ~$3,800 for bevacizumab for the treatment of NSCLC (99), ~$4,800 for pemetrexed for the treatment of NSCLC (100), ~$3,700 for imatinib for the treatment of chronic myeloid leukemia (101), compared to a significantly reduced cost for carbon-ion therapy for recurrent colorectal adenocarcinoma (102).

Cost-effective analysis of various hypofractionated regimens for different disease sites has been performed. For patients with stage I NSCLC, Shah et al. analyzed the cost-effectiveness of SBRT/SABR versus surgical resection (103). The mean costs and quality-adjusted life expectancies for SBRT/SABR, wedge resection, and lobectomy were calculated. For patients determined to be marginally operable, SBRT/SABR was determined to be the most cost-effective strategy. Mitera et al. compared conventional versus SBRT/SABR for medically inoperable stage I NSCLC (104). Overall survival was the primary effectiveness end point used in their calculations. ICER per life-year gained for SBRT/SABR versus CFRT was $1,120, favoring SBRT/SABR as a cost-effective treatment.

For the treatment of localized prostate cancer, comparisons of the costs associated with IMRT versus SBRT/SABR were determined. Delivery of SBRT/SABR is technically more labor-intensive then IMRT; however, treatment is completed in only five fractions compared to 39–48 fractions required for IMRT. A Markov decision analysis model showed that the mean cost of SBRT/SABR is $22,152 versus $35,431 for IMRT (105). A separate analysis by Sher et al. confirmed an ICER favoring SBRT/SABR compared to IMRT (106). Treatment efficacy, rectal toxicity and impotence, and the potential for unseen late effects due to SBRT/SABR were the critical parameters affecting the outcome of the model, highlighting the importance of longer term follow-up to better determine these variables.

Recent criticisms in regard to the use of particle therapy for the treatment of certain malignancies have been raised (107, 108). The investment costs are considerably higher than those for conventional photon therapy (109). Specifically, comparative evidence is lacking on the safety and efficacy of particle therapy versus alternative therapies.

Grutters et al. utilized Markov models to compare treatment with carbon ions, protons, CFRT, and SBRT/SABR for the treatment of patients with inoperable stage I NSCLC (110). Carbon-ion therapy yielded the most quality-adjusted life years per patient of 2.67. Carbon-ion therapy had the highest probability of being cost-effective at 52%, followed by SBRT/SABR at 47%, proton therapy at 2%, and CFRT at 0%.

Comparison of the cost-effectiveness of IMRT, SBRT/SABR, and protons for the treatment of localized prostate cancer were reported by Parthan et al. (111). They found that assuming that each treatment modality results in equivalent long-term efficacy, SBRT/SABR is more cost-effective in improving quality-adjusted survival compared to IMRT or proton therapy. With longer-term follow-up data, it will be interesting to see how carbon ions would compare in the above analysis. The early efficacy and toxicity suggest that it would compare favorably to SBRT/SABR for treating prostate cancer.

The role of cost-effectiveness for particle therapy was excellently reviewed by Pijls-Johannesma et al. (112). In summary, adequate reimbursement is necessary to support such innovative yet costly treatments. Further incorporation of hypofractionated regimens for particle therapy would allow for a higher capacity of patients treated. Currently, the billing model within the United States reimburses per fraction delivered, therefore, motivating more prolonged treatment regimens to be employed. Protons currently share this same model, thereby limiting its utilization. By potentially promoting reimbursement by treatment course for heavy-ion therapy, promotion of higher capacity by treating more patients with fewer fractions could be adopted.

## Discussion and Future Considerations

As shown in this review, using more hypofractionated regimens for photon therapy have resulted in high control rates with minimal normal tissue toxicity. Stereotactic ablative approaches with X-rays have already become the standard of care for patients with inoperative stage I NSCLC. Promising early clinical results highlight the trend to utilize more hypofractionated treatments for other disease sites.

The potential for the further improvement in treatment outcomes by using particle therapy is also generating excitement. Carbon ions, in particular, are attractive due to their superior physical dose distribution, higher RBE, and increased effectiveness in hypoxic tumors, which is expected to generate clinical gains. A systematic approach has been carried out at NIRS and GSI/HIT to determine the optimal dose fractionation regimens for various disease sites, resulting in the majority of tumors being treated with hypofractionated schemes.

Comparing data for different particle treatments is challenging due to different dose regimens, fractionated regimens, and different RBE calculation for carbon ions in different centers. It has been proposed that in the future more prospective clinical trials are necessary to confirm the theoretical benefit of carbon ions. The studies should focus on treatment-related toxicity in addition to local tumor control and survival. However, there is ongoing debate on the necessity to conduct randomized trials before implementing new technologies (57, 112–114). Some argue that based on the principles of equipoise between photons and particle therapy, randomization of patients between the two arms could be unethical (67). Further discussion on these issues should be addressed in future international working groups. In addition, more research into the costs and the optimal way in which to define reimbursement levels for carbon-ion therapy are critical for more widespread implementation.

## Conclusion

With advances in imaging and treatment delivery techniques, the use of more hypofractionated regimens has become more widely employed. Hypofractionated treatment approaches are highly efficacious and safe for the treatment of certain tumors, more cost-effective compared to conventional fractionated approaches, are more efficient use of clinical resources, and also are more convenient for the patient by greatly reducing their treatment course. By utilizing hypofractionated regimens, the potential clinical advantage of particle therapy could be achieved.

## Conflict of Interest Statement

The authors declare that the research was conducted in the absence of any commercial or financial relationships that could be construed as a potential conflict of interest.
